# Integrative network toxicology and pharmacology reveal that kaempferol alleviates BaP-triggered pulmonary fibrosis via suppressing Ras/COX-2-dependent cellular senescence

**DOI:** 10.3389/fphar.2026.1963570

**Published:** 2026-10-15

**Authors:** Qi Zhao, Dongwei Zhu, Zhichao Wu, Xiaolin Xia, Yehui Li, Aojie Guo, Yong Xu, Xianmei Zhou

**Affiliations:** 1 Department of Respiratory Medicine, Affiliated Hospital of Nanjing University of Chinese Medicine, Jiangsu Province Hospital of Chinese Medicine, Nanjing, Jiangsu, China; 2 School of Chinese Medicine, Nanjing University of Chinese Medicine, Nanjing, China

**Keywords:** benzo[a]pyrene, kaempferol, machine learning, network pharmacology, network toxicology, pulmonary fibrosis

## Abstract

**Background:**

Idiopathic pulmonary fibrosis (IPF) is a chronic, progressive lung disease. Benzo[a]pyrene (BaP), a major constituent of cigarette smoke and air pollution, has been closely associated with pulmonary fibrosis. However, its molecular mechanism remains unclear. The natural flavonoid kaempferol possesses anti-inflammatory and antioxidant properties. However, its effects and mechanisms in BaP-induced pulmonary fibrosis remain unclear.

**Objectives:**

This study aimed to predict potential targets of BaP-induced fibrosis and kaempferol intervention in BaP-induced IPF, and to validate whether kaempferol reverses BaP-induced epithelial senescence and fibrosis via the Ras/COX-2 signaling pathway *in vitro*.

**Methods:**

Network toxicology, network pharmacology, machine learning, and molecular docking were integrated to predict potential targets of BaP-induced fibrosis and kaempferol intervention, followed by GO and KEGG pathway enrichment analyses. Additionally, virtual knockout analysis was performed to explore the regulatory relationships among Ras, COX-2, and cellular senescence. MLE-12 cells were used as an *in vitro* model, and senescence-related indicators were assessed by SA-β-gal staining, Western blot, and ELISA.

**Results:**

KEGG enrichment analysis revealed that these targets were significantly enriched in the Ras signaling pathway. Molecular docking confirmed that kaempferol exhibited good binding affinity for the above target proteins. Cell experiments demonstrated that BaP significantly promoted cellular senescence and activated Ras and COX-2. Kaempferol treatment effectively reversed these changes.

**Conclusion:**

This study reveals that BaP promotes IPF by inducing epithelial cell senescence via the Ras/COX-2 signaling pathway. Kaempferol may exert anti-fibrotic effects by counteracting this pathway-mediated senescence. These findings provide a theoretical basis for the pulmonary fibrosis effects of BaP and support kaempferol as a potential anti-fibrotic agent.

## 1 Introduction

Cigarette smoke (CS) is a complex aerosol consisting of gas and particulate phases and containing over 7,000 chemical components, including more than 40 confirmed carcinogens. The main components include nicotine, carbon monoxide, polycyclic aromatic hydrocarbons (PAHs, e.g., benzo[a]pyrene), and heavy metals (Horiyama et al., 2016). Benzo[a]pyrene (BaP), a representative polycyclic aromatic hydrocarbon (PAH), enters the body via the digestive or respiratory tract and undergoes hepatic biotransformation, ultimately producing benzo[a]pyrene-7,8-dihydrodiol-9,10-epoxide (BPDE). This metabolite can induce DNA structural damage (Gurbani et al., 2013; Bukowska et al., 2022).

Numerous review articles have discussed the carcinogenicity and general toxicological effects of BaP. Meanwhile, a growing number of studies have begun to focus on its potential public health implications. BaP can not only interfere with crosstalk between osteoblasts and osteoclasts (Larsen et al., 2020) but also accelerate the degradation of active vitamin D3 via the AhR/CYP24A1 pathway (Matsunawa et al., 2009), thereby impairing bone health. Coke oven workers exposed to BaP exhibit impaired neurobehavioral function, and the higher the urinary level of 1-hydroxypyrene (a biomarker of PAH exposure), the worse the performance on core neurobehavioral tests (Qiu et al., 2013). In addition, elevated exposure levels can cause neurotransmitter disorders (Niu et al., 2010). These findings indicate that more research is warranted to evaluate the comprehensive effects of BaP exposure on human health.

BaP is ubiquitous in the environment, and mammals can be exposed through various routes, including food, air, soil, and water (Faure et al., 2017; Guerreiro et al., 2016). BaP also exerts toxic effects on the respiratory system, as demonstrated by its induction of apoptosis and senescence in mouse lung cells (Yu et al., 2019). Cellular senescence is an irreversible state of cell cycle arrest, characterized by the upregulation of cell cycle inhibitors (e.g., p21 and p16) and the emergence of the senescence-associated secretory phenotype (SASP) (Mebratu et al., 2023). In idiopathic pulmonary fibrosis (IPF), increasing evidence suggests that senescent cells drive disease progression by promoting inflammation, enhancing extracellular matrix (ECM) deposition, and disrupting immune homeostasis (Jin et al., 2025). Because BaP induces cellular senescence (a key driver of IPF), it may promote pulmonary fibrosis through the senescence pathway.

Kaempferol is a flavonoid compound that serves as a key active ingredient in various Chinese herbal medicines and is also abundant in fruits, vegetables, and other plants. Recent studies show that kaempferol inhibits ECM deposition and ameliorates bleomycin-induced pulmonary fibrosis in mice (Zhang et al., 2024). Furthermore, kaempferol can scavenge free radicals and delay cellular senescence (Cai et al., 2025). However, whether kaempferol counteracts BaP-induced pulmonary fibrosis remains unknown.

This study aims to investigate the alleviating effect of kaempferol on BaP-induced pulmonary fibrosis and to elucidate its protective mechanism involving cellular senescence using network toxicological and pharmacological analysis.

## 2 Methods

### 2.1 Acquisition of disease-related targets

Two IPF transcriptome datasets (GSE47460, GSE53845) were downloaded from the NCBI GEO database. Data processing involved extracting expression matrices from both datasets, merging duplicate gene entries by averaging expression values, and selecting common genes to construct an integrated expression matrix. Batch effects were corrected using the ComBat algorithm, and the correction was validated via PCA.

### 2.2 Chemical composition and target acquisition of BaP

The chemical structure of BaP was retrieved from PubChem. Potential targets were screened using ChEMBL, PharmMapper, SwissTargetPrediction, and SuperPred, restricted to *Homo sapiens*.

### 2.3 Differential gene expression analysis

Limma-based transcriptomic analysis identified DEGs using FDR-adjusted P < 0.05 and |log2FC| > 0.585 (1.5-fold change) thresholds. The results were visualized via the ggplot2 package.

### 2.4 Weighted gene co-expression network analysis (WGCNA)

The WGCNA package was used to perform WGCNA. The data were preprocessed by inter-array normalization and low-variance gene filtering (standard deviation >0.5). For sample quality control, abnormal samples were removed via hierarchical clustering and static tree cut (cutting height = 20,000, minimum cluster size = 10). A soft-thresholding power (referred to as optimalPower) was selected based on a scale-free topology fit (R^2^ > 0.85). A network was then constructed using the topological overlap matrix (TOM) similarity and dynamic tree cutting (deepSplit = 2, minModuleSize = 50). Subsequently, similar modules were merged at a cutting height of 0.25. Module-trait associations were evaluated using Pearson correlation (|R| > 0.5, *P* < 0.05), and hub genes were identified based on intra-module connectivity (kME >0.8). Subsequently, KEGG pathway enrichment analysis was performed on the overlapping targets between hub genes and IPF using the DAVID online database (with species set to *H. sapiens*), and the results were visualized.

### 2.5 Identification of core genes using machine learning

A two-stage modeling process (including feature selection and model construction) was performed on the training set expression data using 11 algorithms. A minimum variable threshold (≥3) was set, and cross-validation was used for hyperparameter optimization. The AUC of each model was calculated on its respective training and testing sets, generating an AUC matrix and a heatmap.

### 2.6 Shapley additive explanations

Given the inherent complexity of machine learning models, SHAP analysis quantified each feature’s contribution to predictive outcomes. A SHAP value is assigned to each feature, allowing an interpretable assessment of its predictive impact.

### 2.7 Identification of disease targets related to BaP

To explore the potential mechanism of BaP-induced IPF, the intersection of BaP and IPF targets was screened, and a Venn diagram was generated. Protein–protein interaction (PPI) analysis was performed using the STRING database, and the results were visualized with Cytoscape 3.9.1. GO functional annotation and KEGG pathway analysis were conducted via the DAVID database (with species set to *H. sapiens*), and the results were visualized.

### 2.8 Virtual knockout analysis

Single-cell transcriptome data GSE135893 was downloaded from GEO, including 10 control samples and 20 IPF samples. Seurat was used for quality control, normalization, dimensionality reduction, clustering, and cell annotation. Batch effects were corrected using Harmony.

This analysis used the GSE135893 dataset. Geneformer V2 was used to perform *in silico* triple knockout of HRAS/KRAS/NRAS in IPF epithelial cells, generating perturbation scores (1 – median_cosine) to measure gene sensitivity to RAS loss. Genes detected in fewer than 20 cells (n_cells <20) were excluded. Coverage-matched percentile correction was applied to eliminate n_cells confounding. Pathway enrichment was performed by GSEA (Hallmark, KEGG, Reactome, GO: BP) and module-level permutation tests (10,000 permutations). IPF disease associations were queried via Open Targets. Perturbation scores do not indicate upregulation or downregulation.

### 2.9 Network pharmacology

The chemical structure of kaempferol was retrieved from PubChem. Potential targets were screened using ChEMBL, SwissTargetPrediction, PharmMapper, and TargetNet, with restriction to *H. sapiens*. To explore the potential mechanism of kaempferol in IPF, the intersection of kaempferol and IPF targets was screened, and a Venn diagram was generated. The STRING database was used for PPI analysis, and Cytoscape 3.9.1 was used for visualization. GO functional annotation and KEGG pathway analysis were performed using the DAVID database (with species set to *H. sapiens*), and the results were visualized.

### 2.10 Molecular docking

Molecular docking was employed to verify the binding of kaempferol to the core gene-encoded protein. The 3D structure of the receptor protein was downloaded from the UniProt and PDB databases. The 3D structure of kaempferol was retrieved from PubChem. Docking and visualization were performed using AutoDock Vina and PyMOL.

### 2.11 Cell culture

The immortalized mouse lung epithelial (MLE-12) cells used in this study were purchased from Procell (Wuhan, China). MLE-12 cells were cultured in DMEM containing 10% FBS at 37 °C in a humidified 5% CO_2_ incubator. Three groups were included: a blank control group, a BaP-only group (1 µM BaP for 24 h), and a combination group (1 µM BaP for 24 h followed by 25 µM kaempferol for another 24 h).

### 2.12 Western blotting

Cells were lysed in RIPA buffer, and protein concentrations were determined using a BCA protein assay kit. The samples were loaded onto an SDS-PAGE gel and transferred to a PVDF membrane. The membrane was blocked with NcmBlot Blocking Buffer, then incubated with the primary antibody overnight at 4 °C. After washing with TBST, the membrane was treated with the secondary antibody for 1 h at RT. The membrane was then washed three times with TBST, and signals were detected using an ECL system. Band intensity was analyzed using ImageJ. All antibodies used are provided in Supplementary Table S1.

### 2.13 ELISA

Cell supernatants were collected and centrifuged to remove debris. The levels of interleukin-6 (IL-6), plasminogen activator inhibitor-1 (PAI-1), matrix metalloproteinase-9 (MMP-9), and C-C motif chemokine ligand 2 (CCL-2) in the supernatant were then measured by ELISA per the manufacturer’s instructions (Nanjing Hengyi Biotechnology, China).

### 2.14 Senescence-associated β-galactosidase staining

Cultured cells were fixed and stained using a senescence-associated β-galactosidase staining kit (Beyotime, China) according to the manufacturer’s instructions.

### 2.15 Statistical analysis

All quantitative data were obtained from at least three independent experiments and are expressed as mean ± SD. Statistical analyses were performed using GraphPad Prism 9.5.1. Unpaired two-tailed Student’s t-test was used for comparisons between groups, with *P* < 0.05 considered statistically significant.

## 3 Results

### 3.1 Screening and identification of BaP targets and IPF-related target genes

#### 3.1.1 Identification of BaP potential targets

PubChem provided the molecular structure of BaP, and we employed four databases (ChEMBL, PharmMapper, SwissTargetPrediction, and SuperPred) for target prediction. After integration and deduplication, a final set of 638 unique potential targets was identified (Figures 1A,B).

**FIGURE 1:**
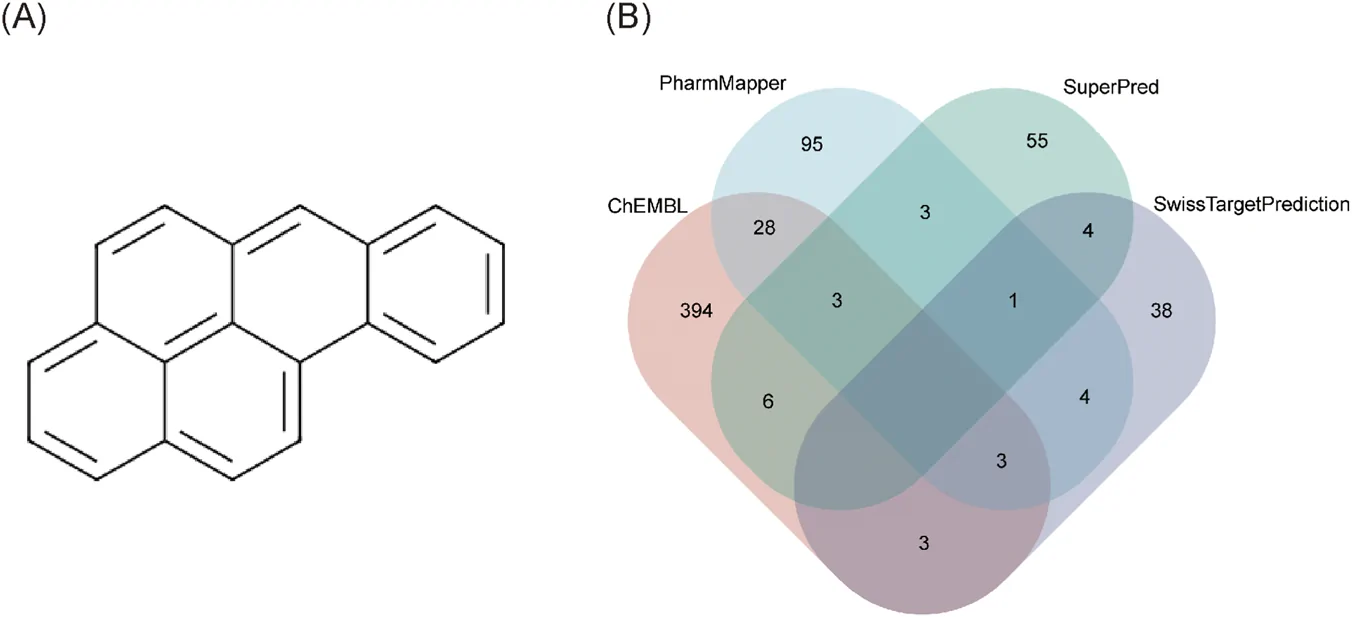
Acquisition of BaP targets. **(A)** Molecular structure of BaP. **(B)** Potential target prediction.

#### 3.1.2 Screening of IPF-related targets

The GSE47460 and GSE53845 datasets were merged and normalized to minimize batch effects. Principal component analysis (PCA) revealed improved data distribution and more pronounced clustering after normalization (Figures 2A,B). 1,424 DEGs were identified in IPF. (Figure 2C).

**FIGURE 2:**
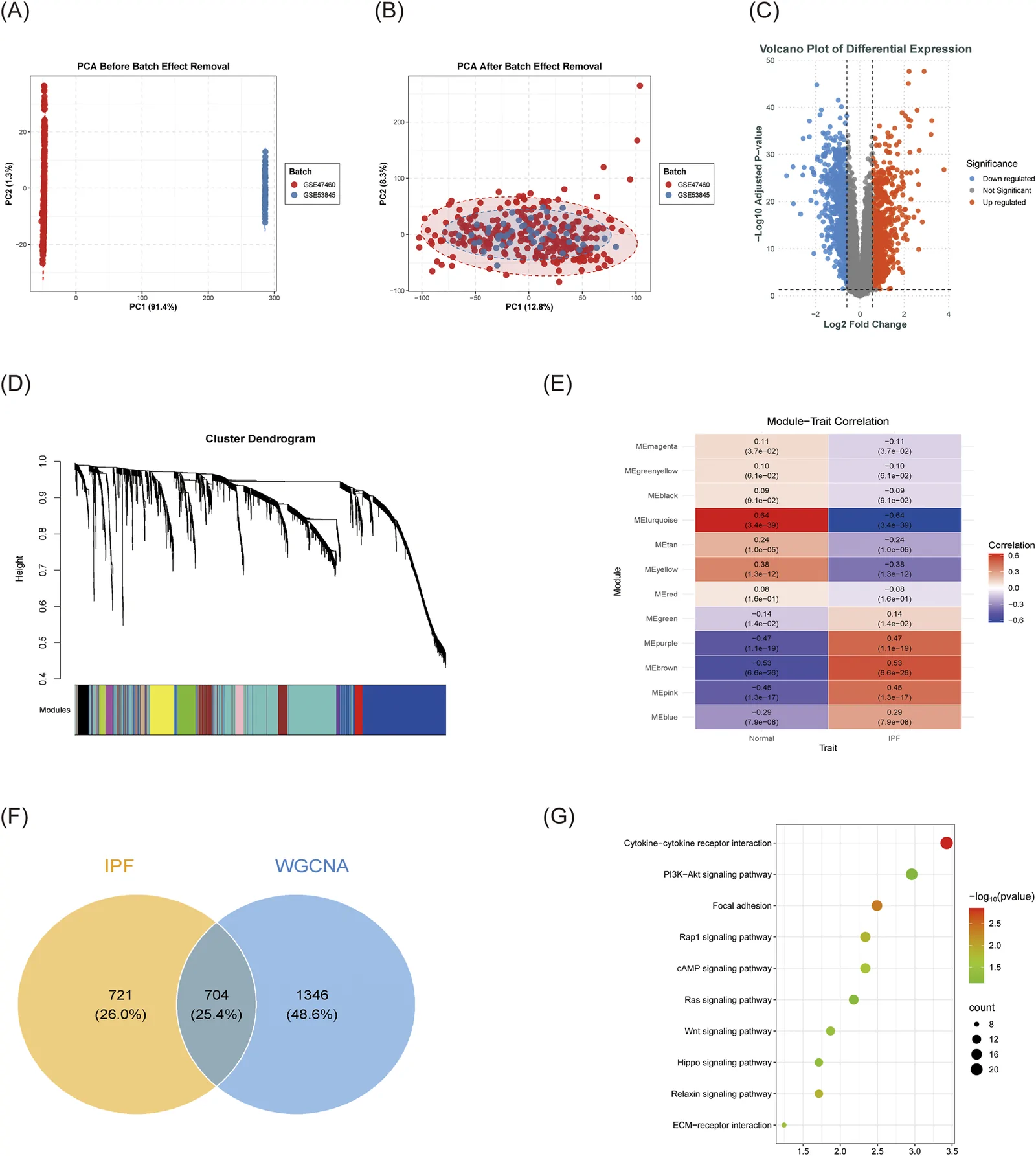
Identification of IPF-related target genes. **(A,B)** The PCA scatter plot. **(C)** The volcano map of DEGs. **(D)** The gene tree diagram of WGCNA. **(E)** The module trait relationship heatmap. **(F)** The Venn diagram shows the overlapping genes between DEGs and WGCNA modules. **(G)** GO and KEGG enrichment analysis of overlapping genes.

In WGCNA, the soft-thresholding power β was evaluated from 1 to 20 to determine the optimal value. Based on the scale-free topology criterion, β = 3 was selected as the optimal soft threshold (R^2^ ≥ 0.8). Using this threshold in hierarchical clustering, we defined 12 co-expression modules, each in a different color (Figure 2D). A significant correlation was observed between the module and IPF traits (*P* < 0.05, Figure 2E). The DEGs were intersected with the WGCNA module genes, and the overlap was visualized using a Venn diagram, yielding a final list of 704 IPF-related genes (Figure 2F). KEGG enrichment analysis of the 704 genes revealed significant enrichment in the Rap1, Wnt, and Ras signaling pathways (Figure 2G).

### 3.2 Identification of intersection targets in BaP and IPF

As shown in Figure 3A, 60 key targets were identified by intersecting BaP-related and IPF-related targets. PPI network analysis was performed on these targets, and they were ranked by node degree. PTGS2 had the highest degree, indicating a core role in the network. The top-ranked genes, including CXCL2, SERPINE1, and IGF1, are all components of the senescence-associated secretory phenotype (SASP) (Figure 3B). Further GO and KEGG enrichment analysis of the 60 targets revealed significant enrichment in signaling pathways associated with core pathological processes of IPF, including the IL-17, TNF, MAPK, and Ras pathways (Figures 3C,D).

**FIGURE 3:**
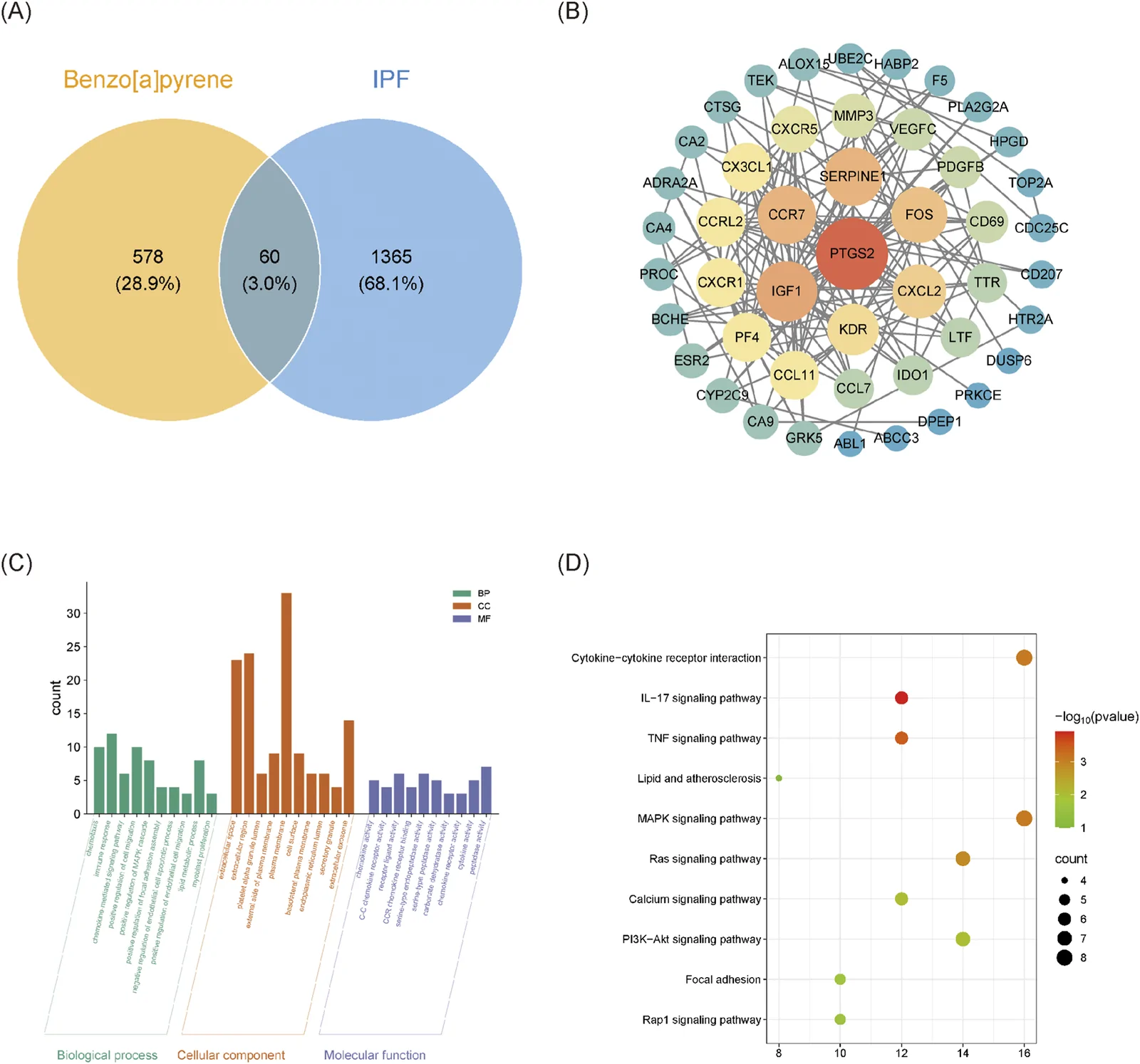
Identification of BaP-related disease targets in IPF. **(A)** The Venn diagram shows 60 overlapping genes between BaP and IPF. **(B)** PPI network diagram of overlapping genes. **(C,D)** GO and KEGG enrichment analysis of overlapping genes.

### 3.3 BaP-induced IPF core gene recognition and machine learning analysis

Using an integrated machine learning method (RF + Enet [alpha = 0.9]), nine core genes (TNC, TEK, CFB, TNNC1, SULT2B1, ABCC3, CXCL2, SERPINE1, and BCHE) associated with BaP-induced IPF were identified from the 60 candidate targets, several of which exhibited good diagnostic performance (AUC >0.85, Figures 4A,B). SHAP analysis identified CXCL2 as the largest contributor to the mean absolute SHAP value. The SHAP beeswarm and force plots further indicated that high CXCL2 expression increases the predicted risk of IPF, whereas overexpression of SULT2B1 and TNNC1 reduces predicted values (Figures 4C–E).

**FIGURE 4:**
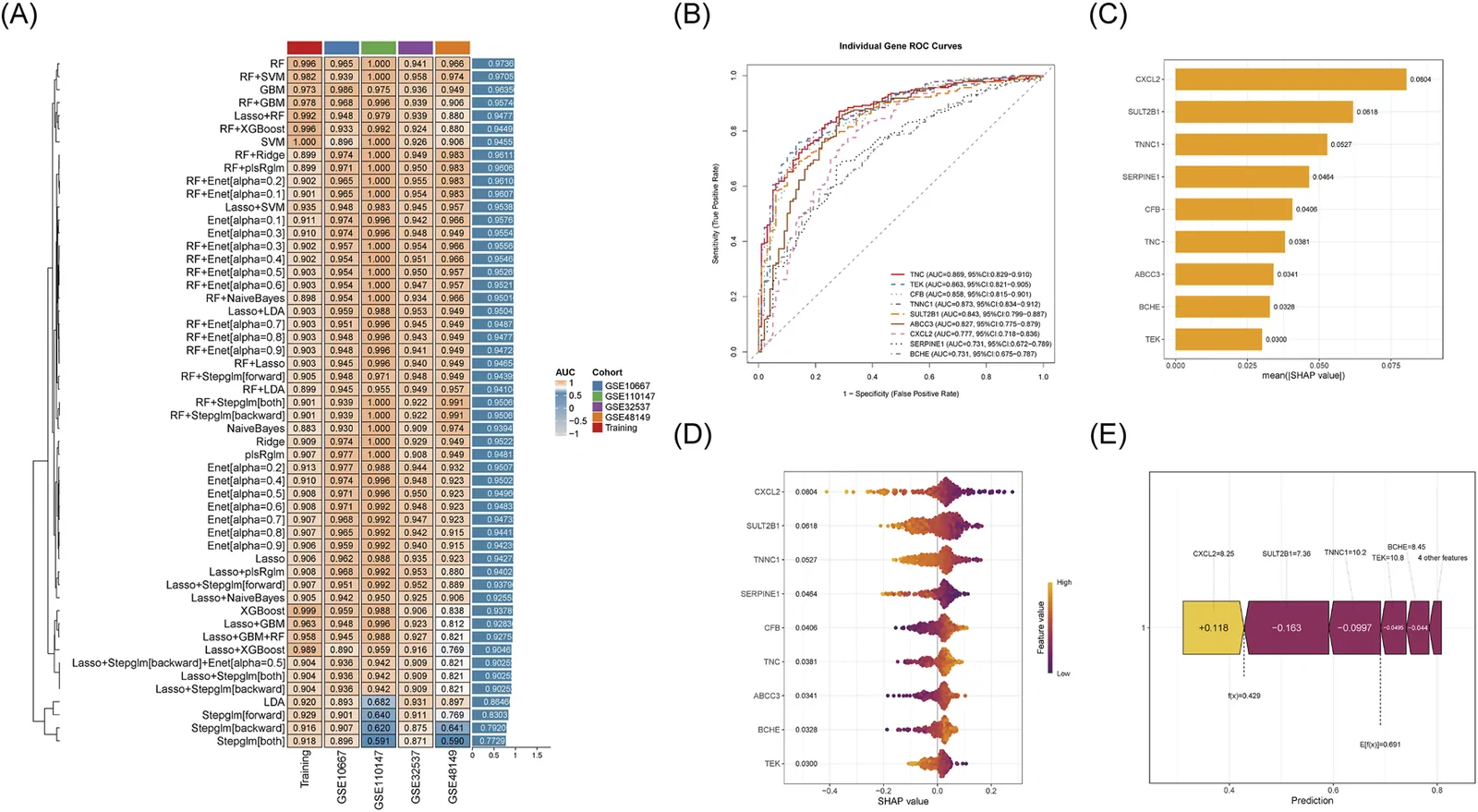
Identification of BaP-induced IPF core genes. **(A)** Different model AUC heatmap across cohorts. **(B)** ROC curves of the nine key genes (TNC, TEK, CFB, TNNC1, SULT2B1, ABCC3, CXCL2, SERPINE1, and BCHE). **(C)** The bar chart of the top genes ranked by feature importance. **(D)** The beeswarm plot of gene expression distribution across conditions. **(E)** The SHAP summary plot of gene contributions to the model.

### 3.4 Virtual knockout analysis

Based on the GSE135893 single-cell transcriptome dataset, we selected pulmonary epithelial cells and performed a triple combined virtual knockout of HRAS/KRAS/NRAS using Geneformer V2 (Figures 5A,B). Among the genes filtered by n_cells ≥20, the top five genes ranked by perturbation sensitivity were FEM1A, XKR8, NCMAP, ZNF699, and SOCS5 (Figure 5C). Focusing on the COX-2/prostaglandin axis and senescence-related pathways, we found that downstream synthases and receptors of prostaglandins exhibited high perturbation sensitivity, and TP53 exhibited extremely high single-gene sensitivity (Figures 5E,F). As shown in Figure 5G, the module-level competitive permutation test showed that the SASP core module experienced significant signal depletion (effect size = −40, P = 0.002).

**FIGURE 5:**
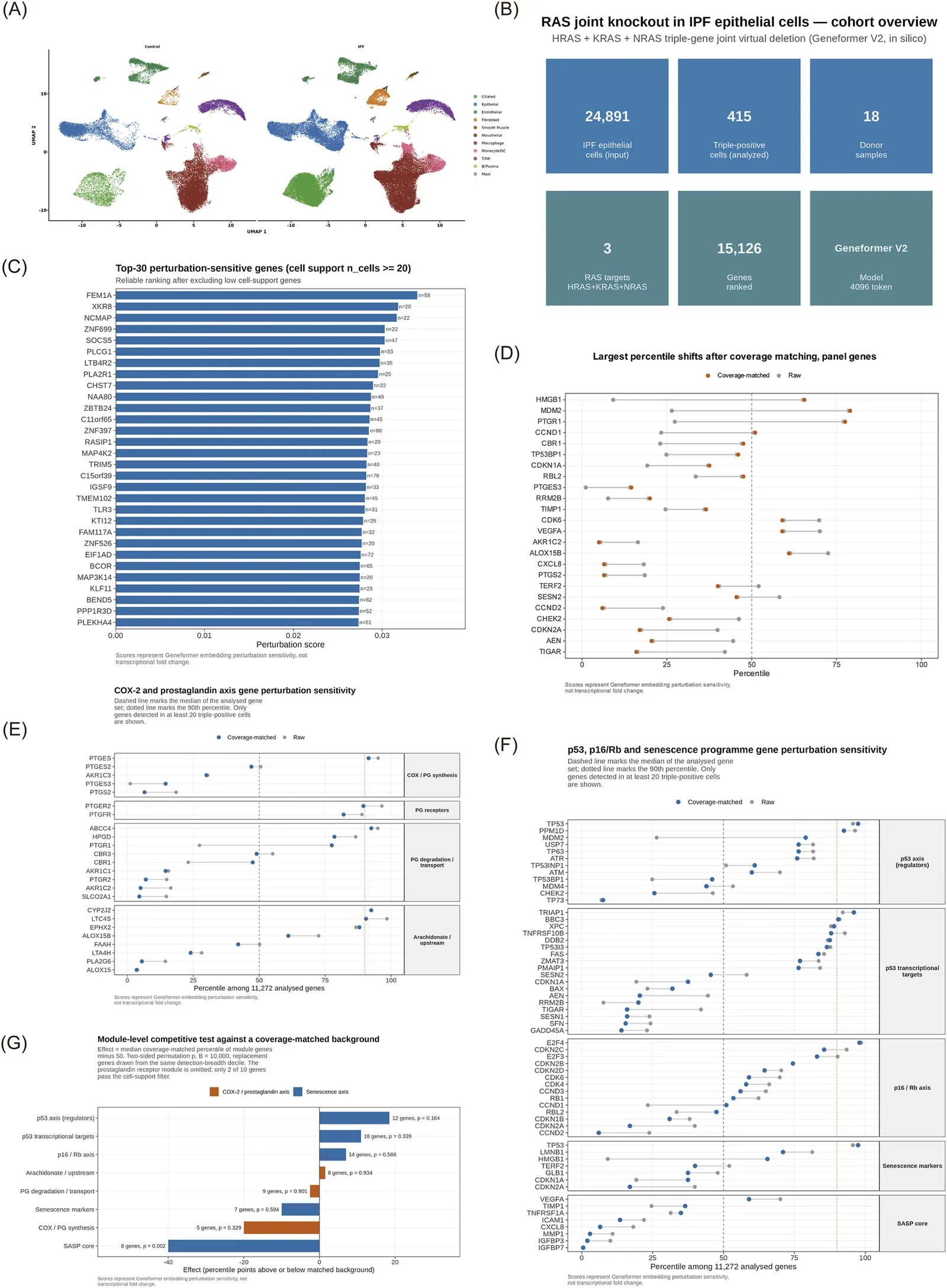
Virtual knockout analysis. **(A)** Cell distribution in normal and IPF lung tissues. **(B)** Cohort overview. **(C)** Top 30 genes with the highest perturbation sensitivity (filtered). **(D)** Percentile displacement before and after correction. **(E)** Perturbation sensitivity of genes in the COX-2/prostaglandin/arachidonic acid axis. **(F)** Perturbation sensitivity of genes in the p53/p21/p16/Rb/SASP axis. **(G)** Competitive permutation test of nine curated modules.

### 3.5 Network pharmacology

A total of 465 kaempferol target genes were obtained by searching the SwissTargetPrediction, PharmMapper, TargetNet, and ChEMBL databases (Figures 6A,B). The intersection of kaempferol targets and IPF targets was identified, and a PPI network was constructed (Figure 6C). Consistent with the previous toxicological results, PTGS2 played a central role in this network. Meanwhile, MMPs and IGF1, which had high degree values, were important components of the SASP (Figure 6D). GO and KEGG enrichment analysis yielded results consistent with those in Figures 3D, 4D, indicating that the Ras signaling pathway plays an important role not only in BaP-induced pulmonary fibrosis but also in kaempferol treatment of IPF (Figure 6F).

**FIGURE 6:**
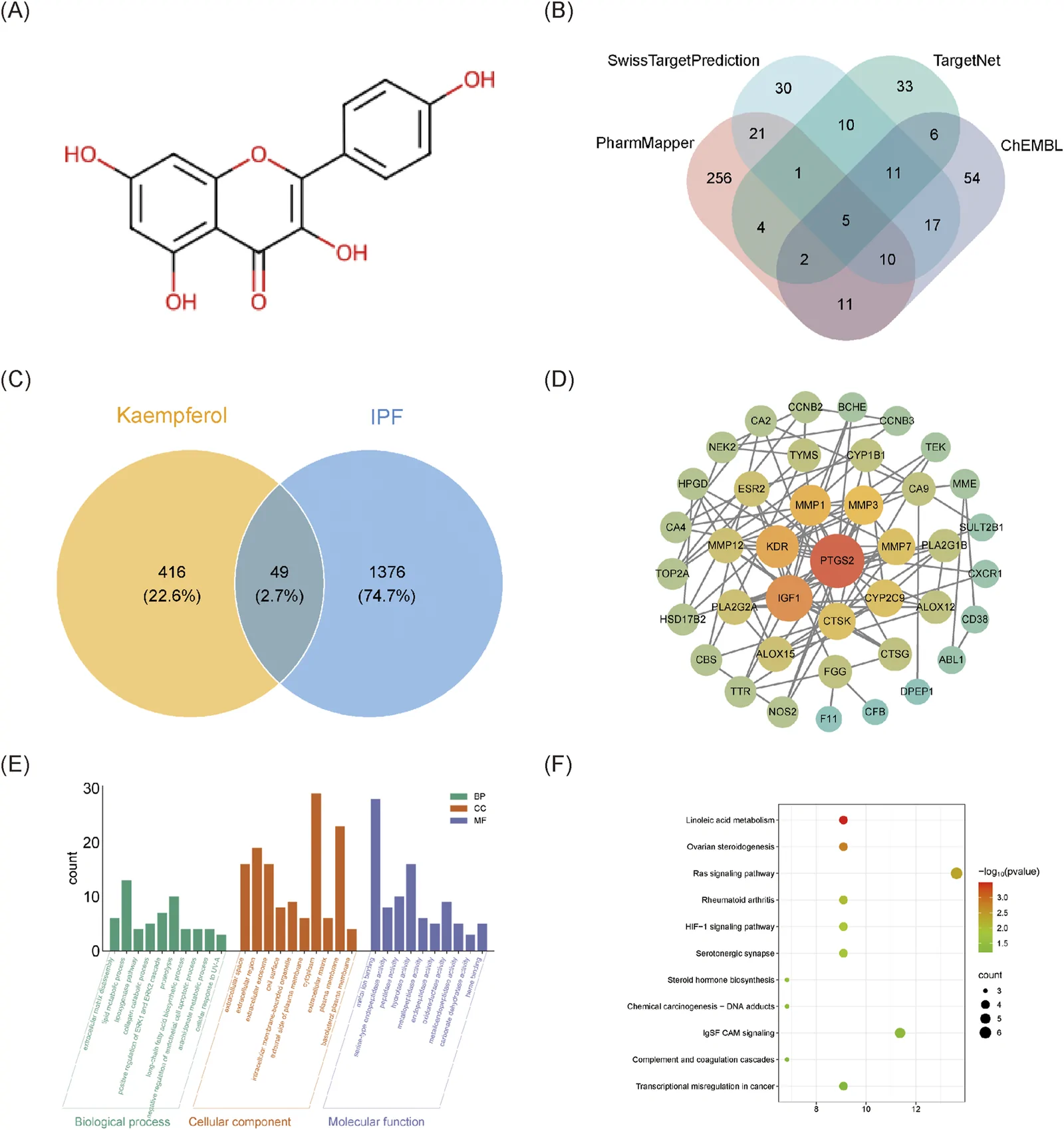
Results of pharmacological analysis of the kaempferol network. **(A)** Molecular structure of kaempferol. **(B)** Prediction of targets from databases. **(C)** The Venn diagram shows 49 overlapping genes between kaempferol and IPF. **(D)** PPI network diagram of overlapping genes. **(E,F)** GO and KEGG enrichment analysis of overlapping genes.

### 3.6 Molecular docking analysis of core genes

Molecular docking was used to investigate the binding of kaempferol to the target proteins: the nine core genes from machine learning (TNC, TEK, CFB, TNNC1, SULT2B1, ABCC3, CXCL2, SERPINE1, and BCHE) and PTGS2 identified in the PPI network. Docking predicted that kaempferol binds to all 10 proteins with binding energies below −5 kcal/mol, indicating potential interactions (Supplementary Table S2). These predictions provide a structural basis for understanding how kaempferol interacts with these proteins (Figures 7A–J).

**FIGURE 7:**
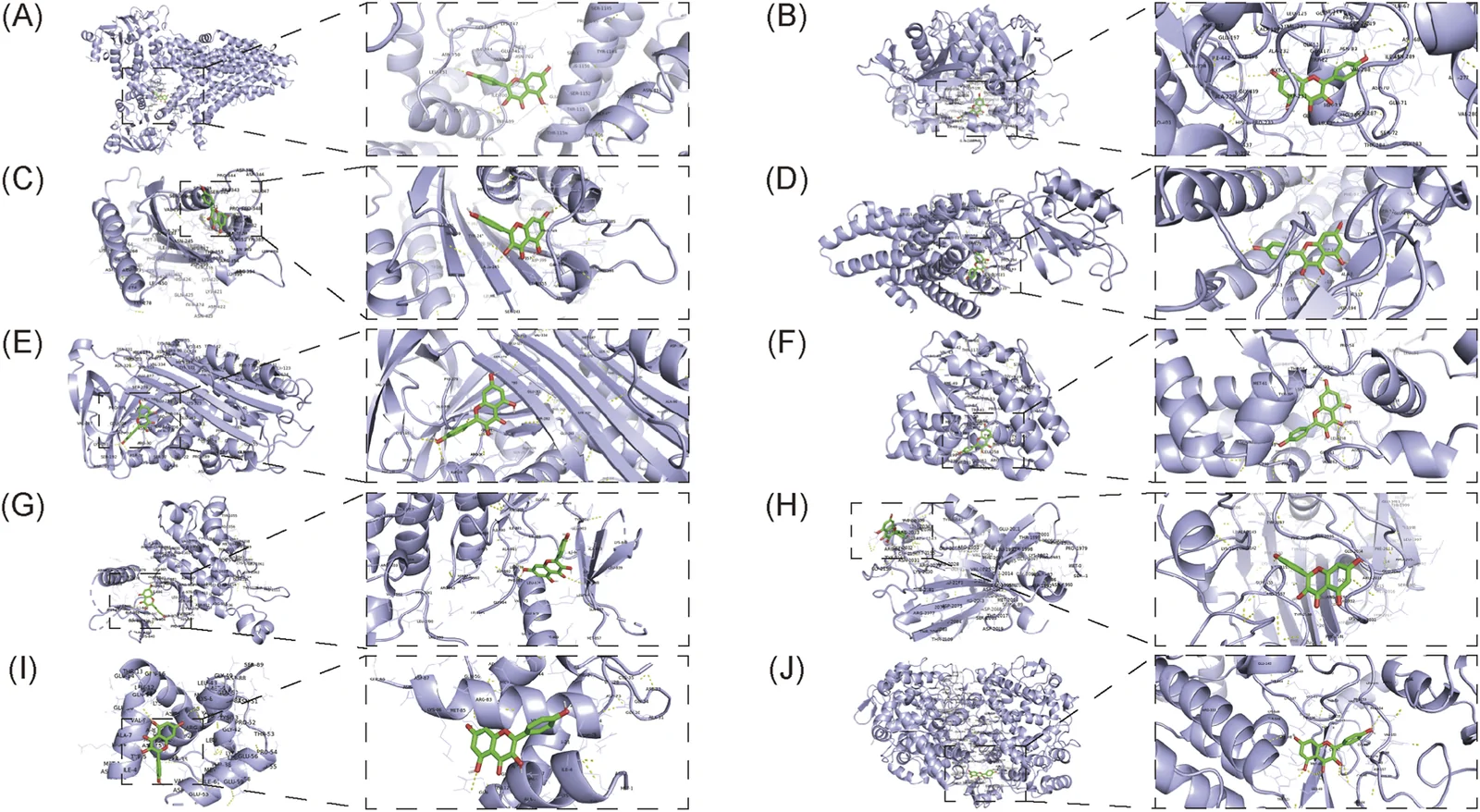
Molecular docking validation. Molecular docking of kaempferol with the ten target proteins: **(A)** ABCC3, **(B)** BCHE, **(C)** CFB, **(D)** CXCL2, **(E)** PTGS2, **(F)** SERPINE1, **(G)** SULT2B1, **(H)** TEK, **(I)** TNC, and **(J)** TNNC1.

### 3.7 BaP promotes pulmonary fibrosis by promoting cellular senescence

Cellular senescence is a stable state of terminal proliferative arrest. Senescent cells typically exhibit a SASP, which includes cytokines, chemokines, proteases, and growth factors (Cao et al., 2025). Both network toxicology and network pharmacology PPI analyses revealed the presence of SASP-related genes (Figures 3B, 6D). Thus, cellular senescence may be involved in the alleviation of BaP-induced pulmonary fibrosis by kaempferol. ELISA was used to detect several SASP factors. We observed that BaP induced SASP-related factors in MLE-12 cells, whereas kaempferol reversed this effect (Figure 8A). Increased SA-β-gal activity is closely associated with cellular senescence. Staining results showed that MLE-12 cells treated with BaP exhibited higher β-galactosidase activity, whereas kaempferol inhibited this enzyme activity (Figure 8B). p53, p21, and p16 play important roles in cellular senescence. Western blot results demonstrated that kaempferol inhibited BaP-induced protein expression of p53, p21, and p16 (Figures 8C,D).

**FIGURE 8:**
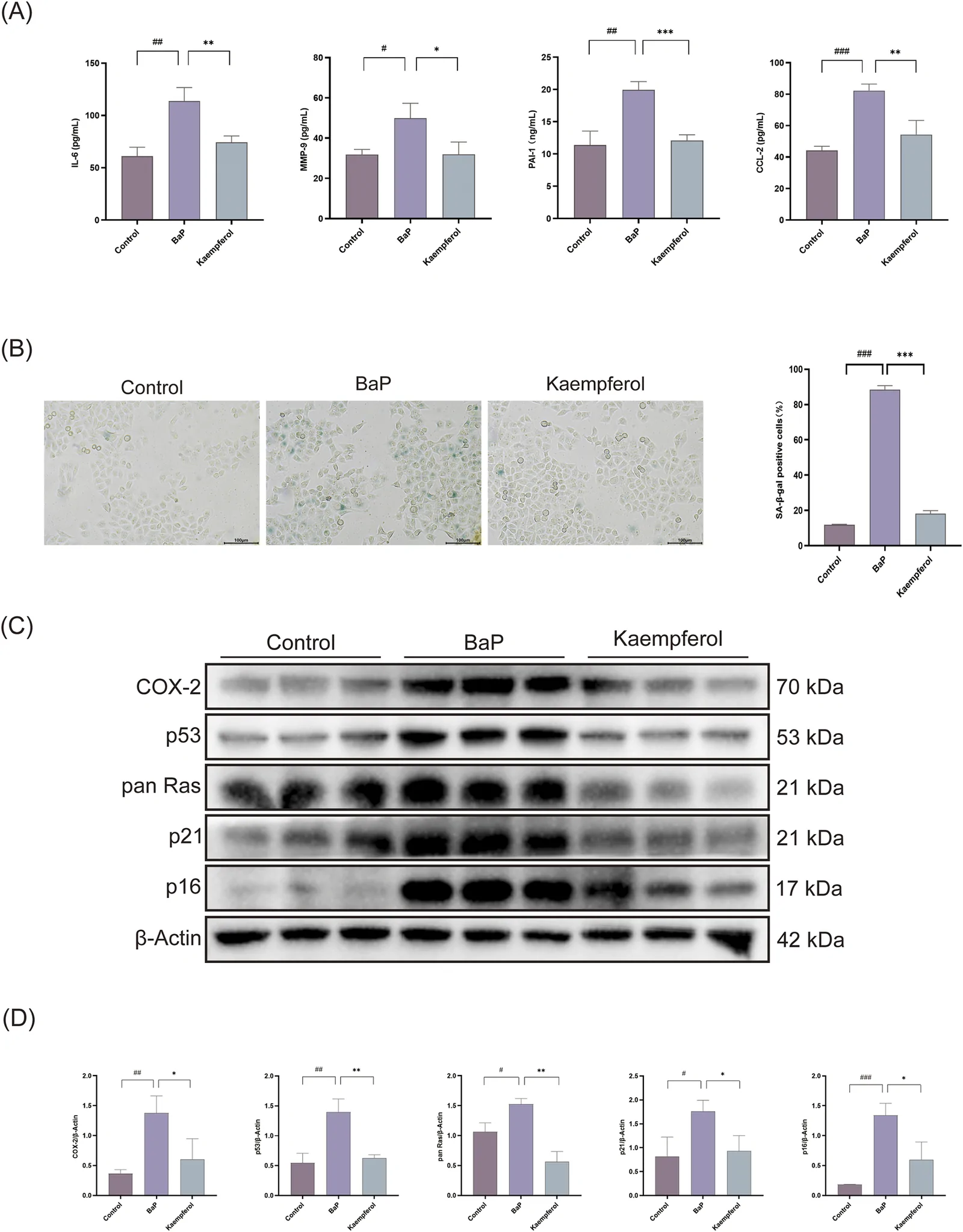
Kaempferol alleviates cellular senescence by suppressing the Ras/COX-2 pathway. **(A)** ELISA analysis of SASP factors. **(B)** β-Galactosidase staining of MLE-12 cells in three groups: control (untreated), BaP (24 h exposure), and kaempferol (BaP exposure followed by kaempferol treatment). **(C,D)** Western blot analysis of COX-2, pan-Ras, p53, p21, and p16 in MLE-12 cells from the same three groups. β-Actin served as a loading control. All experiments were performed with at least three independent replicates. Unpaired two-tailed Student’s t-test was used for analysis. Significance is denoted as ns (not significant), *p < 0.05, **p < 0.01, and ***p < 0.001, ^#^ *p* < 0.05, ^##^ *p* < 0.01, and ^###^ *p* < 0.001, with all comparisons made against the control group.

## 4 Discussion

Pulmonary fibrosis (PF) is a chronic, irreversible lung disorder marked by progressive fibrotic tissue deposition. Its pathological manifestations include marked fibroblast hyperactivation, tissue remodeling, and aberrant ECM accumulation. Treatment options remain suboptimal, and the median survival time for patients with PF is generally 3–5 years (Fang et al., 2025; Jiang et al., 2025). Epidemiological surveys indicate estimated prevalence rates of PF of 4.5–5.7 per 100,000 people in the Asia-Pacific region, 3.3–25.1 in Europe, and 24.0–29.8 in North America (Golchin et al., 2025). Multiple risk factors can promote IPF progression, including intrinsic and extrinsic factors (e.g., smoking and environmental exposure). Epidemiological data show that smoking is one of the most well-recognized environmental risk factors for IPF, with over 60% of patients having a history of smoking (Bae et al., 2022). BaP is a major constituent of cigarette smoke and a key polycyclic aromatic hydrocarbon in air pollutants. The association between BaP and its metabolites and pulmonary fibrosis has been documented. One study found that combined exposure to BaP and nanoplastics induces the formation of extracellular traps in macrophages through the PI3K-AKT/MAPK pathway via activation of relaxin signaling, thereby exacerbating pulmonary fibrosis (Chen et al., 2026). Another study showed that the BaP metabolite BPDE activates the AhR-Nrf2-p62 signaling axis, inhibits Snail degradation via the autophagy-lysosome pathway, promotes epithelial-mesenchymal transition (EMT), and ultimately leads to pulmonary fibrosis (Li et al., 2024). However, existing research has mostly focused on isolated mechanisms or linear pathways and has not systematically revealed the overall network of exposure–response effects.

Cellular senescence represents a state of permanent proliferative arrest, characterized by increased β-galactosidase activity, a senescence-associated secretory phenotype (SASP), and upregulation of cyclin-dependent kinase inhibitors (e.g., p21 and p16). Previous research has confirmed that bleomycin induces cellular senescence in epithelial cells in mouse models of PF (Fan et al., 2025). Furthermore, oncogenic Ras is widely believed to trigger cellular senescence by activating p53 and p16 (Serrano et al., 1997). PPI network analysis, derived from network toxicology and network pharmacology, revealed that the core genes identified by degree values are important components of the SASP. Meanwhile, cell experiments showed that after BaP intervention in MLE-12 cells, the expression levels of p16, p21, and p53 significantly increased, and the secretion of SASP factors into the cell supernatant also increased. SA-β-gal staining further confirmed that BaP induces cellular senescence in epithelial cells.

Ras is a guanosine triphosphatase (GTPase) that regulates cell proliferation, migration, activation, and differentiation by cycling between the inactive GDP-bound and active GTP-bound states (Mozzarelli et al., 2024). Studies have shown that intratracheal instillation of BaP in rats leads to abnormal Ras activation (Qin and Meng, 2010), and that inhibiting the Ras signaling pathway reduces fibroblast activation and EMT, thereby ameliorating pulmonary fibrosis (Zhang et al., 2023). Sustained activation of Ras and its downstream signals induces epithelial cell senescence (Cagnol and Chambard, 2010; Wang et al., 2025) and contributes to disease pathogenesis. WGCNA results showed that BaP-related target genes were markedly enriched in the Ras signaling pathway. Further network toxicology analysis showed that the potential targets of BaP-induced IPF were also enriched in the Ras pathway. Network pharmacology predictions also showed that potential targets of kaempferol, which antagonize IPF, are enriched in this pathway. Moreover, our experiments showed that BaP induces Ras expression in MLE-12 cells.

Cyclooxygenases (COXs), also known as prostaglandin endoperoxide synthases, catalyze the synthesis of prostaglandins from arachidonic acid. Two COX isoenzymes have been identified: COX-1 and COX-2 (encoded by PTGS2). COX-2 induces fibroblast senescence by producing prostaglandin E2 (Martien et al., 2013). Research has shown that COX-2 is significantly upregulated in bleomycin-induced pulmonary fibrosis in mice, and that inhibiting COX-2 alleviates fibrosis symptoms and suppresses senescence-associated protein markers in epithelial cells (Zhang et al., 2020). Mutant Ras (constitutively active) upregulates COX-2 expression, thereby increasing prostaglandin E2 synthesis and promoting pulmonary fibrosis progression (Maciag et al., 2004; Sun et al., 2024). PPI analysis based on network toxicology and network pharmacology revealed that COX-2 plays a central role. Furthermore, our results showed that BaP induces COX-2 expression in MLE-12 cells.

In the COX-2/prostaglandin axis, virtual knockout revealed that downstream synthases and receptors exhibited high perturbation sensitivity, whereas COX-2 itself showed low sensitivity. In the senescence-related pathways, TP53 displayed exceptionally high single-gene sensitivity, while CDKN1A (p21) and CDKN2A (p16) showed low-to-moderate sensitivity. In contrast, cell experiments confirmed that BaP upregulated Ras, COX-2, p53, p21, and p16 expression, and that kaempferol partially reversed these effects. These findings are not contradictory. The virtual knockout reflects the dependency of gene transcription on the presence of Ras signaling, whereas BaP-induced upregulation may involve post-transcriptional regulation and Ras-independent pathways. Together, these complementary observations support a dual-pathway model in which BaP activates both Ras-dependent and Ras-independent mechanisms to drive epithelial senescence, providing a molecular framework for the pro-senescence effects of BaP.

Kaempferol is a flavonoid compound and an important active ingredient in traditional Chinese medicine. It is also widely present in common plants and foods and possesses anti-inflammatory, antioxidant, and immunomodulatory properties. Previous studies have shown that various flavonoids inhibit Ras activity (Cháirez-Ramírez et al., 2021), and that kaempferol modulates Ras and its downstream signaling cascades, thereby participating in the pathological processes of various diseases. In addition, kaempferol has been shown to inhibit COX-2 (Herrera et al., 2025). Kaempferol has been shown to exert a protective effect against pulmonary fibrosis (Zhang et al., 2024; Mawen et al., 2023; Xue et al., 2025). KEGG enrichment analysis revealed that the Ras signaling pathway was significantly enriched in both BaP-induced pulmonary fibrosis targets and kaempferol intervention targets. In addition, virtual knockdown results suggested a potential regulatory relationship between Ras and COX-2. Based on these findings, we propose that kaempferol may exert anti-fibrotic effects by inhibiting the Ras/COX-2 signaling axis, thereby blocking BaP-induced epithelial senescence. This hypothesis was further supported by cellular experimental data: kaempferol intervention significantly attenuated BaP-induced senescence in alveolar epithelial cells, as evidenced by downregulated expression of p53, p21, and p16, reduced secretion of SASP factors, decreased SA-β-gal staining positivity, and concurrent downregulation of Ras and PTGS2 (COX-2) expression.

This study integrates network toxicology, network pharmacology, virtual knockout, and cell experiments to reveal that BaP drives alveolar epithelial senescence by upregulating Ras and COX-2, while kaempferol reverses this process. Mechanistically, sustained activation of the Ras/COX-2 axis increases p53, p21, and p16 expression, driving senescence and SASP secretion. BaP-induced COX-2 upregulation may involve post-transcriptional regulation and Ras-independent pathways. BaP also directly upregulates p21/p16 through Ras-independent routes. Together, these findings suggest that BaP drives epithelial senescence through both Ras-dependent and Ras-independent mechanisms.

Furthermore, this study utilizes network toxicology to offer a systematic framework for exploring complex toxin–host interactions. We found that BaP may directly or indirectly regulate Ras, providing new insights into the environmental etiology of IPF. Previous studies have shown that kaempferol inhibits bleomycin-induced pulmonary fibrosis, but its mechanism has been primarily attributed to antioxidant or anti-inflammatory effects. This study found that Ras also plays an important role in kaempferol treatment of pulmonary fibrosis, which is consistent with its activation in BaP-induced PF. Based on these findings, we link the therapeutic effect of kaempferol on BaP-induced pulmonary fibrosis to BaP-induced Ras/COX-2-mediated cellular senescence.

This study has several limitations. First, network toxicology and network pharmacology predictions are based on public databases such as ChEMBL, PharmMapper, and SwissTargetPrediction, which may lead to false positives and cannot clearly distinguish whether BaP directly binds to the Ras protein or indirectly regulates its expression. Second, the cell experiments were limited to *in vitro* models (MLE-12 alveolar epithelial cells) and were not validated *in vivo*. Third, the study focused only on alveolar epithelial cells and did not include other cell types (e.g., fibroblasts and macrophages) that play key roles in IPF pathogenesis, so it does not fully reflect complex intercellular interactions. Fourth, the Ras family includes multiple subtypes (e.g., H-Ras, K-Ras, N-Ras), and this study did not distinguish between these subtypes or their downstream effects. Fifth, this study did not perform systematic rescue experiments, which limits the definitive identification of functional targets in BaP-induced fibrosis. Future studies should address these limitations.

## 5 Conclusion

This study comprehensively applied network toxicology, network pharmacology, virtual knockout, molecular docking, and cell culture experiments to preliminarily elucidate the molecular mechanism by which the environmental pollutant BaP induces excessive senescence of alveolar epithelial cells via upregulation of Ras and COX-2, thereby promoting the progression of PF. Meanwhile, research has shown that kaempferol protects against PF by antagonizing BaP-induced Ras/COX-2-mediated cellular senescence. Experimental results showed that BaP significantly upregulated the expression of senescence-associated proteins (e.g., p53, p21, and p16) in alveolar epithelial cells, enhanced SASP secretion, and activated Ras and COX-2. Conversely, kaempferol treatment reversed these changes.

## Data Availability

Publicly available datasets were analyzed in this study. This data can be found here: Gene Expression Omnibus (GEO) database, accession numbers GSE47460, GSE53845, GSE10667, GSE110147, GSE32537, and GSE48149.
