# Supplementary material for: Integrative network toxicology and pharmacology reveal that kaempferol alleviates BaP-triggered pulmonary fibrosis via suppressing Ras/COX-2-dependent cellular senescence
Source: Front Pharmacol. 2026 Oct 15;17:1963570. doi: 10.3389/fphar.2026.1963570 (PMC13716622; doi:10.3389/fphar.2026.1963570)
Supplement: Supplementary file 1 [file Supplementaryfile1.docx]

**Supplementary Table 1**.

Table S1. The reagents used in this study.

| Reagent | Supplier | Catalogue |
| --- | --- | --- |
| COX-2 | Abcam | ab179800 |
| pan Ras | Proteintech | 60309-1-Ig |
| p53 | Abmart | TA0879 |
| p21 | Abmart | T55543 |
| P16 | Abmart | TN23895 |
| Benzo[a]pyrene | MCE | HY-107377 |
| Kaempferol | MCE | HY-14590 |

**Supplementary Table 2.**

Table S2. Binding energy.

| Ligand | Receptor | Bind.energy[kcal/mol] | Ligand | Receptor | Bind.energy[kcal/mol] |
| --- | --- | --- | --- | --- | --- |
| Kaempferol | ABCC3 | -7.6 | Kaempferol | SULT2B1 | -10.6 |
| Kaempferol | BCHE | -9.9 | Kaempferol | TEK | -8.0 |
| Kaempferol | CFB | -8.0 | Kaempferol | TNC | -6.7 |
| Kaempferol | CXCL2 | -9.1 | Kaempferol | TNNC1 | -6.1 |
| Kaempferol | SERPINE1 | -6.6 | Kaempferol | PTGS2 | -9.1 |
